# MDCAN-Lys: A Model for Predicting Succinylation Sites Based on Multilane Dense Convolutional Attention Network

**DOI:** 10.3390/biom11060872

**Published:** 2021-06-11

**Authors:** Huiqing Wang, Hong Zhao, Zhiliang Yan, Jian Zhao, Jiale Han

**Affiliations:** College of Information and Computer, Taiyuan University of Technology, Taiyuan 030024, China; wanghuiqing@tyut.edu.cn (H.W.); yanzhiliang0342@link.tyut.edu.cn (Z.Y.); zhaojian0384@link.tyut.edu.cn (J.Z.); hanjiale0421@link.tyut.edu.cn (J.H.)

**Keywords:** lysine succinylation, feature combination, deep learning, dense convolutional block, convolutional block attention module

## Abstract

Lysine succinylation is an important post-translational modification, whose abnormalities are closely related to the occurrence and development of many diseases. Therefore, exploring effective methods to identify succinylation sites is helpful for disease treatment and research of related drugs. However, most existing computational methods for the prediction of succinylation sites are still based on machine learning. With the increasing volume of data and complexity of feature representations, it is necessary to explore effective deep learning methods to recognize succinylation sites. In this paper, we propose a multilane dense convolutional attention network, MDCAN-Lys. MDCAN-Lys extracts sequence information, physicochemical properties of amino acids, and structural properties of proteins using a three-way network, and it constructs feature space. For each sub-network, MDCAN-Lys uses the cascading model of dense convolutional block and convolutional block attention module to capture feature information at different levels and improve the abstraction ability of the network. The experimental results of 10-fold cross-validation and independent testing show that MDCAN-Lys can recognize more succinylation sites, which is consistent with the conclusion of the case study. Thus, it is worthwhile to explore deep learning-based methods for the recognition of succinylation sites.

## 1. Introduction

Post-translational modification of proteins (PTM) is the process of covalent modification on individual amino-acid residues after mRNA is translated into proteins. There are hundreds of known PTMs, mainly including methylation, acetylation, ubiquitination, and succinylation [1]. As a newly discovered PTM [2], succinylation is the process in which a succinyl donor covalently binds succinyl to amino-acid residues by enzymatic or nonenzymatic means. It mainly happens on lysine residues and participates in multiple life activities through regulating the protease activity and gene expression [3]. After succinylation, protein structure is significantly changed due to the combination of lysine residues and succinyl group with large molecular weight. Furthermore, the charge of lysine residues changes from +1 to −1, resulting in a large charge change and further causing changes in the physicochemical properties of amino acids and the functions of proteins [2,4]. Relevant studies have shown that succinylation can regulate various metabolic processes [5,6], whose abnormalities are closely related to the occurrence and development of multiple diseases, including tumors, cardiometabolic diseases, hepatometabolic diseases, and nervous system diseases [7,8]. Therefore, exploring an effective computational method for predicting succinylation sites can help to reveal the differences of succinylation regulatory mechanisms in normal physiological and pathological mechanisms, thus providing certain theoretical support for disease treatment and the research of related drugs.

At present, more and more researchers have applied computational methods to the prediction of protein PTMs [9,10,11,12,13,14,15,16,17], RNA pseudouridine sites [18], and DNA methylation sites [19]. Machine learning-based methods have been widely used in the prediction of succinylation sites. Xu et al. proposed a predictor, iSuc-PseAAC, which incorporated the peptide position-specific propensity into the general form of pseudo amino-acid composition and used support vector machine (SVM) to predict succinylation sites [9]. Hasan et al. developed a computational tool, Succinsite. It distinguished succinylated and non-succinylated lysine residues using k-spaced amino-acid pairs, binary encoding, and amino-acid index property to represent protein sequences. For classification, a random forest classifier was then applied [10]. The predictor Success, proposed by Lopez et al., effectively utilized the structural and evolutionary information to classify original sequences and used SVM to distinguish succinylation from non-succinylation [11]. Jia et al. incorporated the sequence-coupled information into the general pseudo amino-acid composition to represent original sequences, predicted succinylation sites by fusing a series of individual random forest classifiers, and developed a web server, pSuc-Lys [12]. Ning et al. proposed PSuccE using a combination of binary coding, physicochemical properties, and other characteristics. It used information gain for feature selection and ensemble SVM for the detection of succinylation sites [13]. Dehzangi et al. mapped the protein sequences into position-specific scoring matrix profiles as input and adopted the C4.5 decision tree to predict succinylation sites [14]. These methods have all made contributions to accurately identify succinylation sites. However, traditional machine learning methods require manual extraction of features and careful designation based on data, resulting in the dependency on the database and weakening the generalization ability of the model. Therefore, it is very necessary to explore a new deep learning-based method for the recognition of succinylation sites.

Deep learning technology can automatically learn high-level representations from raw data, which overcomes the shortcomings mentioned above. Therefore, it has been applied widely in many fields such as image processing, natural language processing, and bioinformatics [20,21,22,23]. For predicting succinylation sites, Huang et al. used position-specific amino-acid composition, the composition of k-spaced amino-acid pairs, and a position-specific scoring matrix to characterize original sequences [24]. For feature extraction, two-dimensional convolution was used in their method. Ning et al. merged deep neural network (DNN) and penalized logistic regression (PLR) into a hybrid learning architecture, HybridSucc, with 10 features considered [25]. Thapa et al. adopted one-hot encoding and an embedding layer to encode protein sequences. Two-dimensional convolution was also applied for feature extraction [26]. Moreover, the team explored other deep learning frameworks to identify succinylation sites, including recurrent neural network (RNN), long short-term memory network (LSTM), and a cascading model of LSTM and RNN. These studies enrich the applications of deep learning methods in predicting succinylation sites.

Existing deep learning methods have verified the possibility of using deep neural networks to predict succinylation sites. However, these methods adopted traditional convolutional neural networks (CNNs) to extract features and ignored the information exchange and transmission between high-level and low-level layers of the networks. As an improvement of traditional CNNs, the dense convolutional network [27] connects the inputs of different convolutional layers through dense connection. In this way, the high-level convolutional layers can contain the complementary information passed from the low-level ones, which offsets the shortcomings of traditional CNNs. Therefore, considering the complementarity among the features at different levels, with dense convolutional blocks being the feature extractor, can reduce information loss and further learn feature representation with higher quality. Moreover, the max-pooling operation in traditional CNNs selects the local optimal feature to realize certain feature optimization, which means that other local features will be directly discarded. However, the discarded local features also carry important information that helps to predict succinylation sites. Therefore, to utilize these discarded features adequately, we introduce the convolutional block attention module (CBAM) [28]. It learns the differences in importance of different features at both channel and spatial levels, which can realize adaptive optimization of features and further improve the representation ability of the network to identify more succinylation sites.

In this paper, we propose a multilane dense convolutional attention network, MDCAN-Lys, to predict lysine succinylation sites. Considering that succinylation of proteins leads to changes in protein structures and physicochemical properties of amino acids, dense convolutional blocks were adopted to extract sequence information, physicochemical properties of amino acids, and structural properties of proteins to construct feature space. CBAM was applied to weight feature maps and rank the importance of features to achieve adaptive refinement. The refined features were then used as input of a softmax classifier to predict succinylation sites. To verify the predictive performance of MDCAN-Lys, we divided the dataset into a training set and independent test set for 10-fold cross-validation and independent testing, respectively. The experimental results show that, compared with existing methods, the proposed model can effectively learn the abstract pattern of succinylation and identify more succinylation sites. The case study further demonstrates that our model MDCAN-Lys can be used as a powerful tool to assist in the identification of succinylation.

## 2. Materials and Methods

The prediction of lysine succinylation sites can be abstracted as a binary classification problem, i.e., each lysine residue can be classified as having or not having succinylation modification on the residue [29]. In this problem, we took lysine K in the original sequences as the center and cut them into sequence fragments with length L = 2*n* + 1, that is, there were *n* amino acids on both the left and the right sides of lysine K. For sequence fragments containing fewer than L amino acids, we filled them with pseudo amino acids (represented by ‘-’). Each sequence fragment is a piece of data. After numerical vectorization of these sequence fragments from three aspects of sequence information, physicochemical properties of amino acids, and structural properties of proteins, three different characteristics were obtained as the input of the model, MDCAN-Lys. The training set was then used to train the model. Finally, the trained model was used for the prediction of the independent test set and further analysis.

### 2.1. Dataset Collection and Preprocessing

We collected and downloaded the latest experimentally verified lysine-succinylated protein data from the Protein Lysine Modification Database (PLMD) [30]. Considering that, while generating the protein structural property indices, the SPIDER3 server [31] cannot process protein sequences containing nonstandard amino acids, we manually deleted these sequences. High sequence homology can cause model deviation and CD-HIT [32] can be used to remove redundant protein sequences. Therefore, we used CD-HIT with the threshold of 0.3 to strictly screen the protein sequences to ensure their quality. After that, we totally retained 3085 protein sequences and randomly selected 10% (309 sequences) of them as an independent test set. The remaining sequences were used as a training set. The specific information is shown in Table 1.

### 2.2. Information Encoding

The appropriate features of protein sequences or samples play very important roles in the prediction of PTM sites [33]. After the succinylation of proteins, their structures and the physicochemical properties of their amino acids will be changed. Therefore, after extracting sequence information, we further considered physicochemical properties of amino acids and structural properties of proteins to get more abundant vectorization of sequence fragments.

#### 2.2.1. Sequence Information

One-of-21 encoding was used to encode sequence information of the peptide chains, which is a discrete representation with value 1 at the index corresponding to the amino acid in the peptide and 0 at all other positions [29]. For example, the one-of-21 encoding of a sequence fragment ‘MKGLTLNCLG’ is [[000000000010000000000][000000001000000000000]…[000001000000000000000]]. Thus, for a sequence fragment with length L, an L × 21-dimensional vector representation would be obtained after one-of-21 encoding.

#### 2.2.2. Physicochemical Properties

Atchley factors [34] were used to encode the physicochemical properties of amino acids. Each amino acid was represented by five Atchley factors, namely, polarity, codon diversity, secondary structure, molecular volume, and electrostatic charge. For pseudo amino acids, we set the values of all the five factors to 0 (see Table S1, Supplementary Materials, for details). For motifs of length L, we obtained corresponding values according to the correspondence between amino acids contained in them and Table S1. As an example, the corresponding vector representation of a fragment ‘MKGLTLNCLG’ is [[−0.663, −1.524, 2.219, −1.005, 1.212][1.831, −0.561, 0.533, −0.277, 1.648][−0.384, 1.652, 1.330, 1.045, 2.064]…[−1.019, −0.987, −1.505, 1.266, −0.912][−0.384, 1.652, 1.330, 1.045, 2.064]]. Therefore, a motif of length L can be represented by an L × 5-dimensional vector.

#### 2.2.3. Structure Information

We used SPIDER3 [31] to generate information about protein structural properties, including secondary structure (α-helix (ph), β-strand (pe), γ-coil (pc)), local backbone torsion angles (φ, ψ, θ and τ), and accessible surface area (ASA, please see Table S2, Supplementary Materials, for details). As shown in Table S2 (Supplementary Materials), according to the amino-acid composition of the motifs, the corresponding values were found to form the structural characteristic vector representation. For example, a sequence fragment ‘MKGLTLNCLG’ can be represented as [[0.000, 1.000, 0.000, −91.666, 130.708, 113.914, −146.738, 135.508][0.360, 0.594, 0.046, −86.062, 98.634, 109.944, −153.800, 126.646]…[0.132, 0.838, 0.029, 74.432, 7.934, 104.937, −97.450, 40.540]]. Thus, for motifs of length L, L × 8-dimensional vectors would be obtained to represent structure information.

### 2.3. MDCAN-Lys Architecture

**Figure 1:**
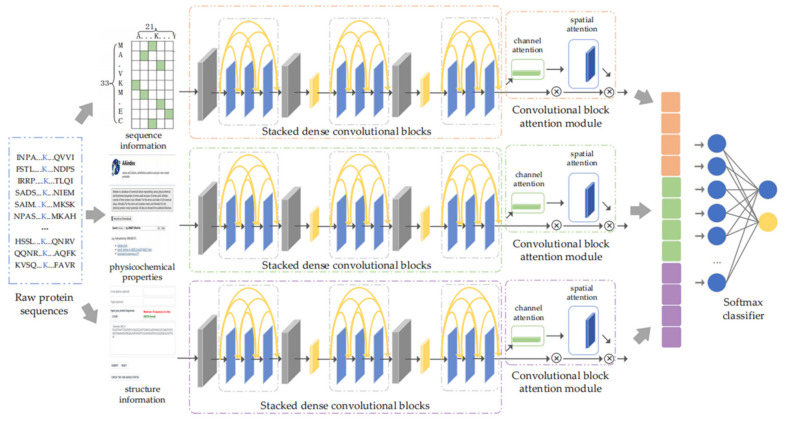
The proposed architecture.

In this paper, a multilane dense convolutional attention network was proposed to learn the potential mechanism of lysine succinylation. The direct fusion of various information before feature learning causes mutual interference, weakens the quality of characteristics, and further influences the effectiveness of feature extraction. Accordingly, we introduced the design idea of a multilane network [35,36,37] and constructed three submodules, i.e., sequence module, physicochemical property module, and structure module. Each module adopted stacked dense convolutional blocks [27] for feature extraction to reduce information loss by considering the complementary characteristics between low-level and high-level convolutional layers. Then, the stacked dense convolutional blocks were followed by CBAM [28] to enhance useful information flow and generate advanced features. Finally, the advanced features obtained from three submodules were fused and fed into a softmax layer to make the final predictions [38]. The model architecture is shown in Figure 1.

#### 2.3.1. Dense Convolutional Network for Feature Extraction

In this part, considering the advantages of multilane network, we constructed a sequence module, physicochemical property module, and structure module. For each module, we applied a dense convolutional network for feature extraction to reduce information loss during feature propagation. The specific implementation process is described below (taking the sequence module as an example).

First, before the application of dense convolutional blocks, a one-dimensional convolutional layer was used to extract features. It took the one-of-21 encoding vectors of motifs with length = L as input, and then generated low-level feature maps of sequence information through the convolution operation, as shown in Equation (1).

(1) $$X^{0}=\sigma (I*W+b),$$

where I denotes the one-of-21 encoding vectors of motifs, and W and b are the weight matrix and bias, respectively. They are trainable parameters during the model training. σ is the exponential linear unit (ELU) activation function [39]. $X^{0}$ represents the low-level feature maps generated by the one-dimensional convolutional layer.

Then, a dense convolutional block was used to extract information from the low-level feature maps $X^{0}$. The dense convolutional block was composed of several one-dimensional convolutional layers with incrementing number of convolutional kernels. Each convolutional layer received the information from previous convolutional layers in the same dense convolutional block as input and generated a high-level feature representation of the sequence information. Taking the l-th convolutional layer in the dense convolutional block as an example, its calculative process would be as shown in Equation (2).

(2) $$X^{l}=\sigma \left(\left[X^{0};X^{1};\ldots ;X^{l-1}\right]*W^{\prime }+b^{\prime }\right),$$

where $X^{l-1}$ denotes the feature maps generated by the (l−1)-th convolutional layer in the dense convolutional block, [.] refers to the concatenation operation along the feature dimension, $W^{\prime }$ and $b^{\prime }$ are weight matrix and bias, which are trainable parameters during model training, σ is the ELU activation function [39], and $X^{l}$ refers to the feature maps generated by the l-th convolutional layer in the dense convolutional block. Thus, the output of a dense convolutional block is the concatenation along the feature dimension of low-level feature maps $X^{0}$ and feature maps generated by each convolutional layer in the dense convolutional block, i.e., $\left[X^{0};X^{1};\ldots ;X^{l}\right]$.

Finally, we used a transition layer for convolution and activation operation on the output of the dense convolutional block. The process of the transition layer is shown in Equation (3).

(3) $$X=\sigma ([X^{0};X^{1};\ldots ;X^{l}]*W^{\prime \prime }+b^{\prime \prime }),$$

where $W^{\prime \prime }$ and $b^{\prime \prime }$ refer to weight matrix and bias, respectively, σ denotes the ELU activation function [39], and X denotes the output of the transition layer. Then, to reduce the dimension of the feature maps and the risk of overfitting, the average-pooling operation was used on the output of the transition layer.

In the sequence module, stacked dense convolutional blocks were composed of multiple identical dense convolutional blocks in series, so as to extract and generate the advanced features of sequence information, $X^{seq}$. Here, we set the number of dense convolutional blocks to three (please see Table S3, Supplementary Materials, for details). Similarly, the physicochemical property module and structure module also generated corresponding advanced features $X^{atc}$ and $X^{stru}$.

#### 2.3.2. CBAM for Adaptive Feature Optimization

Considering that different features have different importance, we introduced CBAM after the dense convolutional network of each module to weight feature maps and enhance useful information flow, thereby further improving the discriminant ability of the network [28]. CBAM is a simple but effective attention module of feedforward convolutional neural network, which is composed of a channel attention module and spatial attention module. Given the input feature, CBAM inferred corresponding attention mapping along two independent dimensions of channel and space to realize adaptive feature optimization. The implementation process is described below.

**Figure 2:**
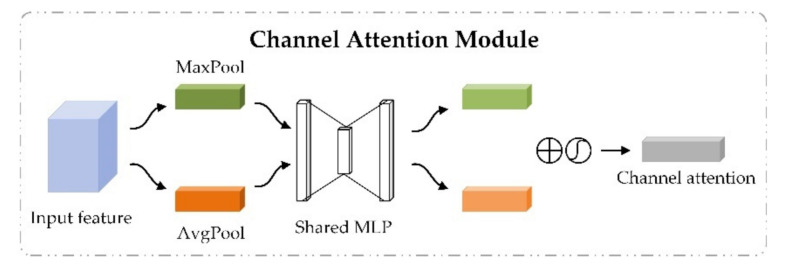
The channel attention module in convolutional block attention module.

The channel attention module highlights important features by setting importance scores for different channel features. Taking the sequence module as an example, the channel attention module took the advanced feature $X^{seq}$ generated by the stacked dense convolutional blocks as input. Through the average-pooling and max-pooling operations, the spatial feature information for $X^{seq}$ was aggregated from global and local perspectives to generate two different spatial context descriptors. The two descriptors were then simultaneously fed into a shared fully connected layer to generate channel attention maps. The channel attention weights were obtained after element-wise summation operation and activation operation of the two generated channel attention maps, as shown in Figure 2. The output features of the channel attention module were obtained through element-wise multiplication between channel attention weights and the input advanced feature $X^{seq}$. The calculative process is shown in Equation (4).

(4) $$X_{c}^{seq}=F(X^{seq},\sigma (MLP(AvgPool(X^{seq}))+MLP(MaxPool(X^{seq})))),$$

where $AvgPool(X^{seq})$ and $MaxPool(X^{seq})$ represent average-pooling and max-pooling operations on the advanced feature $X^{seq}$, respectively, MLP represents the shared fully-connected layer, σ refers to the sigmoid function, $\sigma (MLP(AvgPool(X^{seq}))+MLP(MaxPool(X^{seq})))$ denotes the channel attention weights obtained by the channel attention module, F(.) refers to the element-wise multiplication between the input feature $X^{seq}$ and channel attention weights, and $X_{c}^{seq}$ is the output of channel attention module, which is also the intermediate feature of CBAM.

**Figure 3:**
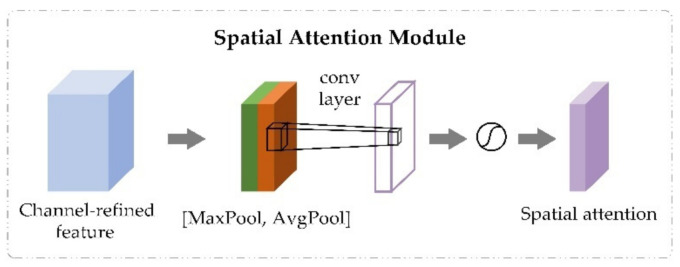
The spatial attention module in convolutional block attention module.

The spatial attention module took the output feature $X_{c}^{seq}$ of the channel attention module as input and generated spatial attention maps using interspatial relationship of features. Unlike the channel attention module, while calculating the spatial attention, the input feature $X_{c}^{seq}$ was compressed first from the channel level. Then, the average-pooling and max-pooling operations were carried out along the channel axis to generate feature descriptors. The feature descriptors were concatenated to generate an efficient descriptor. After that, a convolutional layer was applied to generate spatial attention weights on the descriptor. The process is shown in Figure 3. The output of the spatial attention module was obtained through the element-wise multiplication operation between spatial attention weights and the input feature $X_{c}^{seq}$. The calculative process is shown in Equation (5).

(5) $$X^{\left(seq\right)}=F(X_{c}^{seq},\sigma (f^{7\times 7}([AvgPool(X_{c}^{seq});MaxPool(X_{c}^{seq})]))),$$

where $\left[AvgPool(X_{c}^{seq});MaxPool(X_{c}^{seq})\right]$ denotes average-pooling and max-pooling operations along the channel axis and the concatenate operation, $f^{7\times 7}$ denotes a convolution operation with the filter size of 7 × 7 [28], σ is the sigmoid function, $\sigma (f^{7\times 7}([AvgPool(X_{c}^{seq});MaxPool(X_{c}^{seq})]))$ refers to the spatial attention weights obtained by the spatial attention module, F(.) is the element-wise multiplication between the input feature $X_{c}^{seq}$ and the spatial attention weights, and $X^{\left(seq\right)}$ is the output feature of spatial attention module, which is also the final output of CBAM.

Through the process described above, the sequence module got the weighted advanced feature, $X^{\left(seq\right)}$. Similar to the sequence module, the corresponding weighted advanced features of the physicochemical property module $X^{\left(atc\right)}$ and structure module $X^{\left(stru\right)}$ were also obtained through CBAM. Finally, the weighted advanced features of three modules were connected in series to obtain a fusion feature X for classification. In this paper, the softmax classifier was used to predict succinylation sites. The softmax layer took the fusion feature X as input and obtained the predicted categories of the samples after weighted summation and activation operations. The specific process is shown in Equation (6).

(6) $$P(y=i|x)=\frac{\exp(W_{i}^{S}*X+b_{i}^{S})}{\sum_{i=1}^{2}\exp(W_{j}^{S}*X+b_{j}^{S})},$$

where $W_{i}^{S}$ and $W_{j}^{S}$ are weight matrices, $b_{i}^{S}$ and $b_{j}^{S}$ are bias terms, and P(y=i|x) denotes the probability of sample x being predicted to be class i. While predicting succinylation sites, it has i∈{0,1}. Moreover, for each sample x, the prediction class of the softmax classifier is the category with a higher probability value.

### 2.4. Model Training

In this study, our deep learning model was implemented using Keras 2.1.6 and TensorFlow 1.12.0. While training the model, we adopted dropout [40], early stopping strategy, and L2 regularization to prevent overfitting to further ensure the effectiveness of the model. For solving the data imbalance, we adopted the method of class weight and set the ratio of positive samples and negative samples to 10.9:1. In this way, the model could learn the sequence mechanism from succinylated samples, thus increasing the influence of positive samples and improving the ability of the model to recognize succinylation sites. Additionally, during model training, we used cross-entropy as the cost function and the Adam algorithm [41] to optimize the objective function. To ensure the stability of the training process, we set the learning rate and batch size to 0.0001 and 1000, respectively.

### 2.5. Performance Evaluation

Several statistical measures were considered to evaluate the performance of the proposed model and other predictors. They were sensitivity (*Sn*), specificity (*Sp*), accuracy (Acc), Matthew’s correlation coefficient (MCC), and geometric mean (Gmean). The definitions are as follows:

(7) $$Sn=\frac{TP}{TP+FN},$$

(8) $$Sp=\frac{TN}{TN+FP},$$

(9) $$Acc=\frac{TP+TN}{TP+TN+FP+FN},$$

(10) $$MCC=\frac{TP\times TN-FP\times FN}{\sqrt{\left(TP+FP)\times (TP+FN)\times (TN+FN)\times (TN+FP\right)}},$$

(11) $$Gmean=\sqrt{\frac{TP\times TN}{\left(TP+FN)\times (TN+FP\right)}},$$

where *TP*, *TN*, *FP*, and *FN* represent true positives, true negatives, false positives, and false negatives, respectively. *Sn* was used to evaluate the accuracy in identifying succinylation sites. *Sp* revealed the predictor’s ability to recognize non-succinylation sites. Acc measured the number of correctly classified lysine residues. When the positive and negative samples were unbalanced, MCC could be used to measure the classification quality of a binary classifier [42]. Gmean is another indicator for measuring the quality of a classifier, which herein measured the balance between the classification performance of succinylated and non-succinylated sites [43,44]. We also used the area under the receiver operating characteristic (ROC) curve (AUC) and the area under the precision recall rate (PR) curve (AUPR) to further access the overall performance of the model. 

To accurately evaluate the performance of the proposed model on each statistical index, k-fold cross-validation and independent testing were adopted. For k-fold cross- validation, we took k = 4, 6, 8, and 10 for experiments.

## 3. Results and Discussion

### 3.1. Selection of Window Size

**Figure 4:**
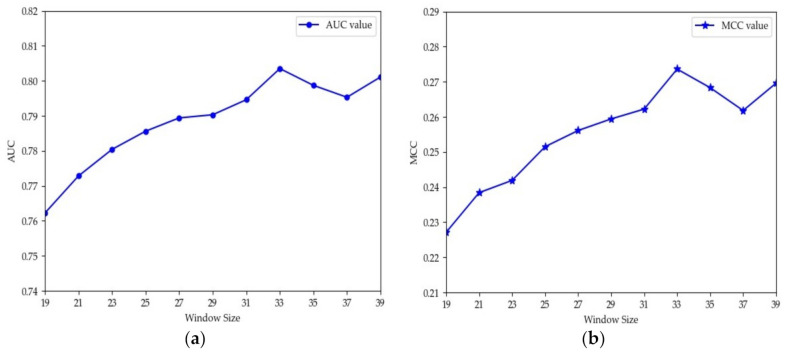
AUC and MCC values vary with different window sizes for 10-fold cross-validation: (**a**) the values of AUC under different window sizes; (**b**) the values of MCC under different window sizes.

**Figure 5:**
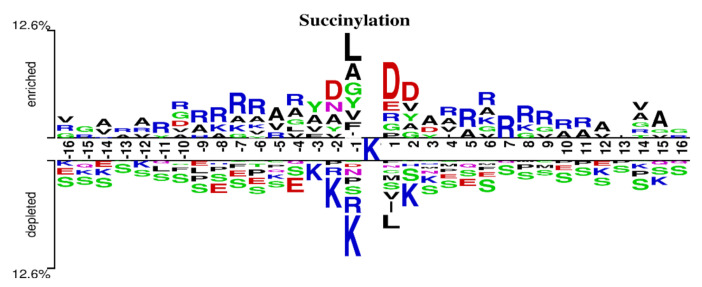
Two Sample Logos of positive and negative samples with L = 33.

The choice of window size is closely related to the performance of methods. Different methods usually have different appropriate window sizes. Jia et al. and Lopez et al. set L to 31 [12,45]. Thapa et al. proved through experiments that 33 was the optimal length [26]. Hasan et al. set the window size to 41 for their experiments [46]. Ning et al. set L to 21 and 51 [13,47]. To explore the optimal window size of the proposed model in this paper, we performed 10-fold cross-validation using the training set in Table 1. Considering that the sequences with length greater than 40 may form structural domains [48], which may extract more structural information and potentially cause deviation of the model, we set L to values from 19 to 39 for experiments and recorded the values of AUC and MCC obtained using different window sizes. The results are shown in Figure 4. From Figure 4, we can see that the values of AUC and MCC increased until L = 35. Moreover, when L was 33, the values both reached the maximum. Thus, a window size of 33 was adopted in this paper. To verify if MDCAN-Lys was overfitting with L = 33, we drew the training/validation loss/accuracy curve for the 10-fold cross-validation on the training set shown in Table 1 (for details, please refer to Figure S1, Supplementary Materials). According to the curve trend, we concluded that the model proposed in this paper was not overfitted. To further verify the rationality of the selected length, we analyzed the positive and negative samples with L = 33 using Two Sample Logos [49], as shown in Figure 5. From Figure 5, we can see that the differences in amino-acid distribution between positive and negative samples were great at positions greater than −16 or less than 16, which provides certain biological support for the choice of 33 as the optimal window size [46,47,50].

### 3.2. Comparison with Existing Methods

To evaluate the performance of the proposed model, we compared MDCAN-Lys with other existing methods for predicting succinylation sites. Six representative methods were considered, namely, iSuc-PseAAC [9], SuccinSite [10], pSuc-Lys [12], HybridSucc [25], DeepSuccinylSite [26], and iSuc-PseOpt [45]. Among them, iSuc-PseAAC, SuccinSite, pSuc-Lys, and iSuc-PseOpt are classical traditional machine learning-based methods for recognizing succinylation sites. The methods they use include SVM, random forest, and integrated random forest. HybridSucc adopts 10 feature representations, such as position-specific scoring matrix and accessible surface area, to characterize protein sequences and merges DNN and PLR into a hybrid-learning architecture. Since these methods only provided web servers, we evaluated them only on the independent test set in Table 1. DeepSuccinylSite is a leading application of the deep learning-based methods to predict succinylation sites. It performed one-hot encoding and embedding encoding on the protein sequences and used CNN for feature extraction. For the experiments based on the training set in Table 1, to compare the performance of multilane dense convolutional attention network and traditional CNNs, we reproduced DeepSuccinylSite using both one-hot and embedding encoding to represent sequences and recorded the results of 10-fold cross-validation, as shown in Table 2. To further evaluate the robustness of the proposed model, we performed k-fold cross-validation with k = 4, 6, and 8. The results are also shown in Table 2.

From Table 2, we can see that our method obtained higher *Sp*, Acc, Gmean, and AUC, indicating that our proposed model had a good performance on the training set. However, the MCC and AUPR of our method were relatively low (the same applies to the results of k-fold cross-validation when k = 4, 6, and 8 except for the value of Gmean when k = 6). This is because the class weight adopted in our method focused more on forcing the model to learn the potential mechanism of positive samples during training. While preprocessing the dataset, DeepSuccinylSite implemented undersampling on the benchmark dataset and constructed a balanced subset for model training. However, MCC and AUPR are usually sensitive to the imbalance of datasets [42,44]. Furthermore, it is worth noting that, although DeepSuccinylSite ^b^ had a higher *Sn*, its *Sp* was 55.62%, indicating that, while DeepSuccinylSite ^b^ could recognize most succinylation sites, there were still many non-succinylated sites identified as succinylated sites. In the same 10-fold cross-validation, our method achieved *Sn* of 66.81% with higher *Sp*, which indicates that MDCAN-Lys could simultaneously recognize most non-succinylation and succinylation sites. In addition, for both DeepSuccinylSite ^b^ and our method, the values of *Sn* and *Sp* were somewhat confrontational, which is consistent with those described in [44]. On the whole, our proposed multilane dense convolutional attention network could extract features with higher quality to learn the differences in potential mechanism between succinylated and non-succinylated sequences.

To analyze the robustness of the proposed model, we further performed k-fold cross-validation setting k = 4, 6, and 8, the results of which are shown in Table 2. From Table 2, we can see that there was no significant fluctuation among the index values in the four cases, especially for MCC, Gmean, AUC, and AUPR. The congruence of results for k-fold cross-validation indicates the promising performance of MDCAN-Lys and that our model is robust.

To further compare the predictive ability of MDCAN-Lys with other methods, we uploaded the independent test set in Table 1 to the web servers provided by these methods. According to the prediction results obtained from the web servers, we calculated corresponding values of Acc, *Sn*, *Sp*, MCC, and Gmean (for DeepSuccinylSite, we trained the model based on the training set and made predictions on the independent test set in Table 1). For these methods, we were unable to report their AUC and AUPR values, because there are no independent tools provided. The experimental results are shown in Table 3.

As can be seen from Table 3, for the independent testing, our model MDCAN-Lys obtained higher values of *Sn*, Gmean, AUC, and AUPR, which indicates the effectiveness of our method. This comparison considered four traditional machine learning methods (iSuc-PseAAC, iSuc-PseOpt, SuccinSite, and pSuc-Lys) and two deep learning methods (HybridSucc, and DeepSucinylSite); as such, we discuss them independently. Compared with the deep learning-based methods, MDCAN-Lys achieved the highest values of Acc, *Sn*, *Sp*, MCC, Gmean, AUC, and AUPR. That is, compared to traditional DNNs and CNNs, the cascading model of dense convolutional blocks and convolutional block attention module helped to extract more advanced and useful information to recognize succinylation sites. For methods based on traditional machine learning, iSuc-PseAAC, iSuc-PseOpt, SuccinSite, and pSuc-Lys all obtained higher Acc (except for iSuc-PseOpt) and *Sp* values, but lower *Sn*, especially for iSuc-PseAAC, iSuc-PseOpt, and pSuc-Lys. However, *Sn* represents the percentage of all positive cases that were predicted as positive examples, thereby measuring the ability of the classifier to identify positive examples [50]. This suggests that these predictors paid much attention to the negative samples, leading to failure in accurately identifying more true succinylation sites. Moreover, the higher Acc values obtained by these methods were partly due to the recognition of more non-succinylation sites. In contrast, our method obtained the highest *Sn* (70.32%) among all the methods with an *Sp* of 73.23%, indicating that our method could not only identify most non-succinylation sites, but also had a better ability to identify the true succinylation sites. In the biological field, the goal is to find as many succinylation sites as possible, which means that predictors with higher *Sn* are more suitable for experimental verification [50]. This demonstrates the effectiveness of our method. Moreover, it is worth noting that the web servers of these methods were pretrained using most sequences. Therefore, it is possible that some sequences in our uploaded test set were used to train the predictors and, thus, biased their results. In this case, the promising results of our method suggest that it is worth exploring the application of multilane dense convolutional attention network in predicting succinylation sites.

### 3.3. Ablation Experiments

To verify the importance of the used three feature representations and the necessity of each module in MDCAN-Lys, we performed ablation experiments from two aspects (feature combination and model architecture) by 10-fold cross-validation using the training set shown in Table 1.

#### 3.3.1. Feature Combination Ablation Experiment

In this paper, we combined sequence information, physicochemical properties of amino acids, and structural properties of proteins to characterize original sequences. To prove that all the three features help to predict succinylation sites, we conducted 10-fold cross-validation on the basis of different single features or feature combinations. The results are shown in Table 4. For the first column in Table 4, Feature ^a^ denotes sequence information, Feature ^b^ denotes physicochemical properties of amino acids, and Feature ^c^ denotes structural properties of proteins.

Different columns in Table 4 refer to different single features or feature combinations. The last column is the strategy that adopted three features synchronously, which was the feature combination used in this paper. We recorded the values of Acc, MCC, AUC, and AUPR under various cases. As shown in Table 4, the proposed model performed best overall when all three feature representations were used. Although the value of Acc was not the highest in this case, it was still higher than that obtained using only the sequence information. Therefore, on a whole, we chose these three characteristics to characterize protein sequences. This suggests that, using the sequence information, the quantitative representation of each amino acid by secondary structure, local backbone torsion angle, and accessible surface area can characterize the discrete information and important continuous information of the local structure and properties of amino acids, thus helping the model to better learn the underlying mechanism of succinylation [17,29].

#### 3.3.2. Model Architecture Ablation Experiment

**Figure 6:**
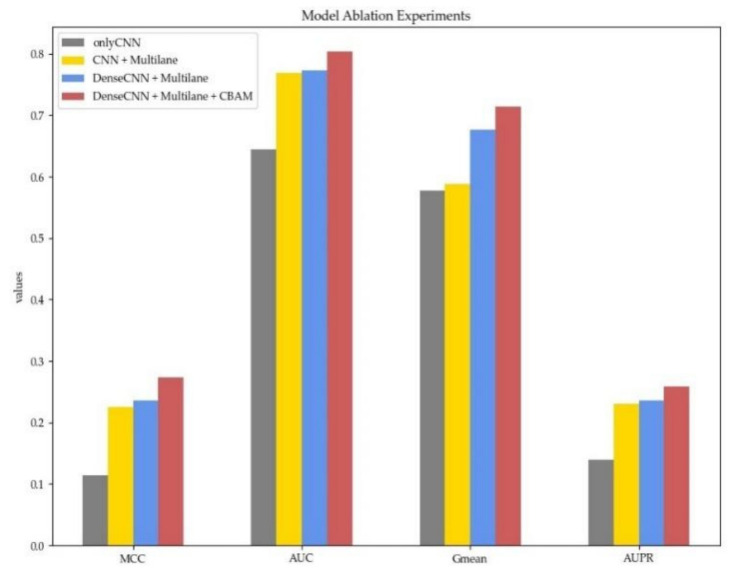
The 10-fold cross-validation performance with different model architectures. Different colors represent different model configurations. Red denotes our proposed model.

To verify the necessity of each part in MDCAN-Lys, we designed an ablation experiment based on the model architecture. We defined a benchmark model, which took the concatenation of sequence information, physicochemical properties of amino acids, and structural properties of proteins as input and extracted features by traditional CNNs. Then, other parts were added in turn, including the adoption of the multilane network, the use of dense convolutional network, and the addition of CBAM. The experimental results are shown in Figure 6 (for details, please refer to Table S4, Supplementary Materials).

Different colors in Figure 6 represent different model architectures, with the gray one being the baseline model mentioned above. The yellow one shows the results of multilane CNN, whose MCC, AUC, Gmean, and AUPR were all improved. The improvement indicates that using a multilane network to extract three features separately can effectively avoid information crosstalk and enable the model to learn feature representations with higher quality. To verify the advantages of dense convolutional network compared with traditional CNNs, we further changed the multilane CNN into a multilane dense convolutional network (the results are shown in blue). From Figure 6, we can see that MCC, Gmean, AUC, and AUPR were all improved, indicating that the dense convolutional network can simultaneously use characteristic information at different levels through dense connection. In this way, it can adequately utilize information flow in the network to further obtain higher-quality abstract representation. Lastly, CBAM was added after each dense convolutional network (the proposed model in this paper). All the indicators were further improved, which shows that, by learning the feature weights on the channel and spatial levels, CBAM can judge “what” and “where” information of a given input sequence is meaningful. In addition, average-pooling and max-pooling used in the two modules of CBAM could consider both the most important local characteristic and the global characteristics, which offsets the information loss caused by using the max-pooling operation only. Furthermore, it enhances the representation ability of the network so that the model can get better performance.

### 3.4. Biological Insights into Succinylation Prediction

To further analyze the predictive results of each classifier on the independent test set, similar to [11,17], we manually counted the predictive results of human proteins in the independent test set (see Table S5, Supplementary Materials, for detailed results). Then, according to their Uniprot Accessions, we searched the Uniprot database (https://www.uniprot.org/, accessed on 9 February 2021) [51] for their important functions and pathways. From Table S5, we can see that MDCAN-Lys could predict most proteins with more than two succinylation sites. These proteins include deoxycytidine kinas (Uniprot Accession P13667), which is related to protein folding and protein secretion, cyclohydrolase (Uniprot Accession P13995), which is concerned with folic acid and one-carbon metabolic processes, peroxisomal multifunctional enzyme type 2 (Uniprot Accession P51659), which is a bifunctional enzyme acting on the peroxisomal beta-oxidation pathway for fatty acids, and leucine-rich PPR motif-containing protein (Uniprot Accession P42704), which plays a role in RNA metabolism in both nuclei and mitochondria. Moreover, MDCAN-Lys showed accurate identification of proteins with two succinylation sites. Some of these proteins include *N*-alpha-acetyltransferase 15 (Uniprot Accession Q9BXJ9), whose activity may be important for vascular, hematopoietic, and neuronal growth and development, ADP-ribose glycohydrolase MACROD1 (Uniprot Accession Q9BQ69), which could be involved in invasive growth by downregulating CDH1 in endometrial cancer cells, methylglutaconyl-CoA hydratase (Uniprot Accession Q13825), which is related to the leucine catabolic process, and 40S ribosomal protein S11 (Uniprot Accession P62280), which plays a role in the nuclear-transcribed mRNA catabolic process. In addition, MDCAN-Lys correctly detected some proteins with only one succinylation site. A few examples are myristoylated alanine-rich C-kinase substrate (Uniprot Accession P29966), which can bind calmodulin and synapsin, and serine/threonine-protein phosphatase PP1-gamma catalytic subunit (Uniprot Accession P36873), which regulates glycogen metabolism, muscle contractility, and protein synthesis. Lastly, there are some proteins whose sites can only be detected by one method. Two of these proteins were phosphatidylethanolamine-binding protein 1 (Uniprot Accession P30086), which acts as a serine protease inhibitor, and NADH dehydrogenase 1 alpha subcomplex subunit 7 (Uniprot Accession O95182), which functions in the transfer of electrons from NADH to the respiratory chain. The results on the independent test set show that our method could identify a large number of succinylation sites.

**Figure 7:**
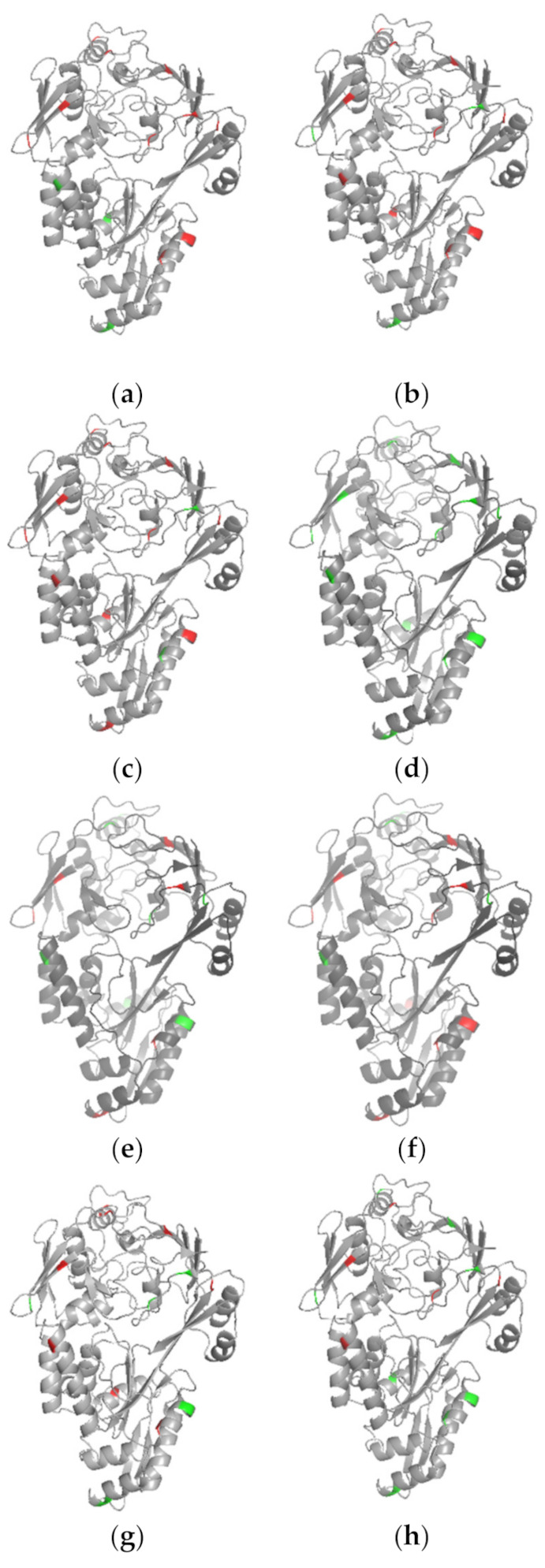
Visualization comparisons of various classifiers on protein P23847: (**a**) iSuc-PsaAAC; (**b**) pSuc-Lys; (**c**) HybridSucc; (**d**) SuccinSite; (**e**) DeepSuccinylSite ^a^; (**f**) DeepSuccinylSite ^b^; (**g**) iSuc-PseOpt; (**h**) our method.

To analyze the predictive performance of each classifier more intuitively, we compared the predicted results of one succinylated protein (P23847) in the independent test set visually, as shown in Figure 7 (see Table S6, Supplementary Materials, for the predicted results). A total of 13 sites were modified with succinylation for the protein. In Figure 7, mispredicted amino acids are shown in red and correctly predicted amino acids are shown in green. We note that SuccinSite predicted all sites correctly. Actually, we checked the training set provided on the web server of SuccinSite and found that protein P23847 was used as one of the training data for model training. Thus, we do not discuss much about SuccinSite in this case. Beyond this, from Figure 7 we can see that MDCAN-Lys predicted the most correct sites, and the prediction accuracy was significantly higher than other predictors, which indicates that the feature combination and model architecture we adopted were reasonable and effective. It is worth noting that different methods did not predict exactly the same correct sites. Additionally, there were still some methods with high accuracy, such as DeepSuccinylSite ^a^ and iSuc-PseOpt. That is, although our method could predict more succinylation sites correctly, all the predictors should be used in a complementary way to obtain more complete outcomes to identify more potential succinylation sites.

## 4. Conclusions

In this paper, we proposed a new model based on deep learning for predicting succinylation sites, MDCAN-Lys. Considering sequence information, physicochemical properties of amino acids, and structural properties of proteins for feature representations, MDCAN-Lys used a multilane dense convolutional network to extract features and a convolutional block attention module to further optimize features. The results of k-fold cross-validation and independent testing showed that MDCAN-Lys can be used as a powerful tool to assist in the recognition of lysine succinylation modification. In addition, the results of ablation experiments based on feature combinations and model architecture indicated that using multilane dense convolutional attention network to extract sequence information, physicochemical properties of amino acids, and structural properties of proteins can help to transform the original sequence fragments into meaningful abstract representations, thereby further helping the model to better complete the lysine succinylation prediction. In the future, we will try to adopt more feature representations (such as a position-specific scoring matrix [16,52,53,54] or protein function features [55]) and explore other deep learning networks (such as a capsule network [56,57] or improved CNN models [58]) for the prediction of succinylation sites.

## Figures and Tables

**Table 1:** Number of positive and negative samples for training set and independent test set.

Dataset Type	Number of Proteins	Positive Samples	Negative Samples
Training Set	2776	5885	64,140
Independent Test Set	309	684	6709

**Table 2:** The k-fold cross-validation performance of our method vs. DeepSuccinylSite (the data are percentages). DeepSuccinylSite ^a^ and DeepSuccinylSite ^b^ denote DeepSuccinylSite with one-hot encoding and embedding encoding. The highest value in each category is shown in italic.

Method	*Sn*	*Sp*	Acc	MCC	Gmean	AUC	AUPR
DeepSuccinylSite ^a^	69.84	68.11	69.87	*38.16*	68.77	75.92	*72.63*
DeepSuccinylSite ^b^	*75.69*	55.62	65.66	32.03	64.80	71.66	68.93
Our Method (k = 4)	63.69	77.48	76.32	26.11	69.76	79.36	24.28
Our Method (k = 6)	60.58	*79.59*	*77.99*	26.48	68.65	79.73	24.96
Our Method (k = 8)	67.26	75.71	74.99	26.80	71.03	79.94	25.33
Our Method (k = 10)	66.81	76.75	75.91	27.36	*71.37*	*80.35*	25.88

**Table 3:** Comparison of MDCAN-Lys with existing predictors using the independent test set (the data are percentages). DeepSuccinylSite ^a^ and DeepSuccinylSite ^b^ represent DeepSuccinylSite with one-hot encoding and embedding encoding. The highest value in each category is shown in italic.

Method	Acc	*Sn*	*Sp*	MCC	Gmean	AUC	AUPR
iSuc-PseAAC	82.34	13.29	*89.23*	2.32	34.44	-	-
iSuc-PseOpt	72.33	31.17	76.45	2.32	48.81	-	-
SuccinSite	*84.20*	58.79	86.74	*34.51*	71.41	-	-
pSuc-Lys	78.39	23.22	83.89	5.47	44.14	-	-
HybridSucc	63.00	39.00	65.40	2.65	50.50	-	-
DeepSuccinylSite ^a^	56.96	68.42	55.79	14.07	61.78	66.75	16.77
DeepSuccinylSite ^b^	62.63	65.35	62.35	16.37	63.83	68.67	17.48
Our Method	72.96	*70.32*	73.23	27.33	*71.76*	*79.03*	*25.24*

**Table 4:** The 10-fold cross-validation performance with different single features or feature combinations (the data are percentages). The highest value in each category is shown in italic.

**Feature ^a^**	√			√	√		√
**Feature ^b^**		√		√		√	√
**Feature ^c^**			√		√	√	√
Acc	68.93	76.64	76.06	75.59	68.24	*77.12*	75.91
MCC	26.67	24.85	4.53	26.74	23.43	26.17	*27.36*
AUC	79.99	78.62	55.55	79.63	77.45	79.04	*80.35*
AUPR	25.70	23.98	10.36	24.62	22.70	24.46	*25.88*

## Data Availability

Not applicable.

## Supplementary Materials

The following are available online at https://www.mdpi.com/article/10.3390/biom11060872/s1, Table S1. The detailed values of 5 atchley factors for all the amino acids. ‘-’ means pseudo amino acid; Table S2. An example for the detailed values of structure information. P(C), P(E), and P(H) are secondary structures. Phi, Psi, Theta, and Tau are local backbone torsion angles. ASA means accessible surface area; Table S3. The 10-fold cross-validation performance with different numbers of dense convolutional blocks on the training set in Table 1 (the data are percentages). The largest value for each indicator is highlighted in italic; Table S4. The 10-fold cross-validation performance with different model architectures (the data are percentages). The largest value for each indicator is highlighted in italic; Table S5. Details on the predictive results of human proteins for different methods on the independent test set. ’Total’ means the total number of succinylated sites of a particular protein. Method a denotes iSuc-PseAAC. Method b denotes SuccinSite. Method c denotes pSuc-Lys. Method d denotes iSuc-PseOpt. Method e denotes HybridSucc. Method f denotes DeepSuccinylSite with one-hot encoding. Method g denotes DeepSuccinylSite with embedding encoding. Method h denotes MDCAN-Lys (our method); Table S6. Details on the predictive results of protein P23847 in the independent test set for different methods; Figure S1. The training/validation loss/accuracy curve of the proposed model MDCAN-Lys by 10-fold cross-validation with window size = 33 on the training set described in Table 1. The blue line is training and the orange line is validation.
