# Supplementary material for: MDCAN-Lys: A Model for Predicting Succinylation Sites Based on Multilane Dense Convolutional Attention Network
Source: Biomolecules. 2021 Jun 11;11(6):872. doi: 10.3390/biom11060872 (PMC8231176; doi:10.3390/biom11060872)
Supplement: Supplementary file 1 [file biomolecules-11-00872-s001.zip › biomolecules-1215875-supplementary.pdf]

MDCAN-Lys: A Model for Predicting Succinylation Sites Based on Multilane Dense Convolutional Attention Network

Huiqing Wang [1], Hong Zhao *, Zhiliang Yan, Jian Zhao, Jiale Han

College of Information and Computer, Taiyuan University of Technology, Taiyuan 030024, China; wanghuiqing@tyut.edu.cn (H.W.); zhaohong0383@link.tyut.edu.cn (H.Z.); yanzhiliang0342@link.tyut.edu.cn (Z.Y.); zhaojian0384@link.tyut.edu.cn (J.Z.); hanjiale0421@link.tyut.edu.cn (J.H.)

* Correspondence: zhaohong0383@link.tyut.edu.cn (H.Z.)

Supplementary Tables

Table S1. The detailed values of 5 atchley factors for all the amino acids. '-' means pseudo amino acid.

Amino acid	FactorI	FactorII	FactorIII	FactorIV	FactorV
A	-0.591	-1.302	-0.733	1.570	-0.146
C	-1.343	0.465	-0.862	-1.020	-0.255
D	1.050	0.302	-3.656	-0.259	-3.242
E	1.357	-1.453	1.477	0.113	-0.837
F	-1.006	-0.590	1.891	-0.397	0.412
G	-0.384	1.652	1.330	1.045	2.064
H	0.336	-0.417	-1.673	-1.474	-0.078
I	-1.239	-0.547	2.131	0.393	0.816
K	1.831	-0.561	0.533	-0.277	1.648
L	-1.019	-0.987	-1.505	1.266	-0.912
M	-0.663	-1.524	2.219	-1.005	1.212
N	0.945	0.828	1.299	-0.169	0.933
P	0.189	2.081	-1.628	0.421	-1.392
Q	0.931	-0.179	-3.005	-0.503	-1.853
R	1.538	-0.055	1.502	0.440	2.897
S	-0.228	1.399	-4.760	0.670	-2.647
T	-0.032	0.326	2.213	0.908	1.313
V	-1.337	-0.279	-0.544	1.242	-1.262
W	-0.595	0.009	0.672	-2.128	-0.184
Y	0.260	0.830	3.097	-0.838	1.512
-	0.000	0.000	0.000	0.000	0.000

Table S2. An example for the detailed values of structure information. P(C), P(E), and P(H) are secondary structures. Phi, Psi, Theta, and Tau are local backbone torsion angles. ASA means accessible surface area.

#	sequence	P(H)	P(C)	P(E)	Phi	Psi	Theta	Tau	ASA
1	M	0.000	1.000	0.000	-91.666	130.708	113.914	-146.738	135.508
2	K	0.360	0.594	0.046	-86.062	98.634	109.944	-153.800	126.646
3	G	0.495	0.417	0.089	-67.886	-28.976	104.914	51.980	34.582
4	L	0.510	0.367	0.123	-80.839	-18.508	104.622	78.073	50.994
5	T	0.518	0.354	0.129	-82.939	-13.558	106.243	81.074	44.509
6	L	0.693	0.209	0.097	-71.644	-31.179	99.516	93.514	41.454
7	N	0.675	0.234	0.091	-73.767	-28.184	99.682	58.869	85.859
8	C	0.608	0.345	0.047	-78.531	-22.289	100.256	57.057	34.127
9	L	0.364	0.596	0.040	-81.739	-13.829	101.870	61.127	49.580
10	G	0.132	0.838	0.029	74.432	7.934	104.937	-97.450	40.540
...	...	...	...	...	...	...	...	...	...
...	...	...	...	...	...	...	...	...	...
...	...	...	...	...	...	...	...	...	...

Table S3. The 10-fold cross-validation performance with different numbers of dense convolutional blocks on the training set in Table1 (the data are percentages). The largest value for each indicator is highlighted in italic.

Dense Blocks	Sn	Sp	Acc	MCC	Gmean	AUC	AUPR
2	63.66	77.15	76.02	25.72	70.05	78.87	24.16
3	*66.81*	76.75	75.91	*27.36*	*71.37*	*80.35*	*25.88*
4	60.29	79.16	77.58	25.65	68.81	79.22	23.88
5	57.95	*80.55*	*78.66*	25.54	68.11	79.07	23.67

Table S4. The 10-fold cross-validation performance with different model architectures (the data are percentages). The largest value for each indicator is highlighted in italic.

Model	MCC	Gmean	AUC	AUPR
only CNN	11.39	57.70	64.38	13.87
CNN + Multilane	22.45	58.82	76.86	23.10
DenseCNN + Multilane	23.61	67.58	77.34	23.52
DenseCNN + Multilane + CBAM	*27.35*	*71.37*	*80.35*	*25.88*

Table S5. Details on the predictive results of human proteins for different methods on the independent test set. 'Total' means the total number of succinylated sites of a particular protein. Method [a] denotes iSuc-PseAAC. Method [b] denotes SuccinSite. Method [c] denotes pSuc-Lys. Method [d] denotes iSuc-PseOpt. Method [e] denotes HybridSucc. Method [f] denotes DeepSuccinylSite with one-hot encoding. Method [g] denotes DeepSuccinylSite with embedding encoding. Method [h] denotes MDCAN-Lys (our method).

Protein	Total	Method[a]	Method[b]	Method[c]	Method[d]	Method[e]	Method[f]	Method[g]	Method[h]
P13667	4	0	3	1	2	4	1	2	3
P13995	3	0	1	1	1	2	1	0	2
O14929	1	0	1	0	0	1	1	0	1
O15355	1	0	1	0	0	0	0	0	1
O43175	2	0	2	1	1	2	1	0	2
O43290	1	0	1	0	1	1	1	0	0
O75521	2	1	0	0	1	0	1	0	0
O76003	1	0	1	0	0	0	1	1	0
O95139	2	0	1	0	0	1	0	0	0
O95182	1	0	0	0	0	0	1	0	0
P0DME0	2	0	1	0	1	1	2	1	0
P16401	1	1	1	0	0	0	1	0	1
P21964	1	0	1	0	0	0	1	1	1
P27824	1	1	1	1	1	0	1	1	1
P29966	1	0	1	0	0	0	0	1	1
P30086	1	0	1	0	0	0	0	0	0
P31943	2	0	2	0	0	1	1	1	2
P36873	1	0	1	0	1	0	1	0	1
P39748	2	0	2	0	1	0	1	1	2
P41227	1	0	1	0	0	0	1	0	1
P42126	2	0	2	1	2	0	0	0	0
P49753	1	0	1	1	1	1	1	1	1
P49755	2	1	2	0	0	0	1	1	2
P62280	2	0	1	0	0	0	0	1	2
Q10713	1	0	1	1	1	0	0	0	1
Q13243	1	0	1	1	1	0	1	1	1
Q13825	2	1	2	0	0	1	1	1	2
Q5JRX3	2	0	1	2	1	0	1	1	1
Q8WV44	1	0	1	1	0	0	1	1	0
Q8WVY7	1	0	1	0	0	0	1	1	1
Q92665	1	0	1	0	0	0	1	1	1
Q9BQ69	2	0	2	1	1	0	1	1	2
Q9BXJ9	2	0	2	0	2	1	1	0	2
Q9UHD1	1	0	1	0	1	0	0	0	1
P51659	7	0	6	2	4	2	2	2	6
P42704	13	1	7	1	4	10	8	5	8

Table S6. Details on the predictive results of protein P23847 in the independent test set for different methods.

Protein/ Methods	Succinylation site/ Predicted succinylation sites	Total/ correctly predicted
P23847	81、89、100、196、227、319、324、364、419、482、514、517、526	13
iSuc-PseAAC	364、419、482	3
SuccinSite	81、89、100、196、227、319、324、364、419、482、514、517、526	13
pSuc-Lys	89、364、517	3
iSuc-PseOpt	89、324、364、517、526	5
HybridSucc	319、517	2
DeepSuccinylSite[a]	100、196、324、419、482、514、526	7
DeepSuccinylSite[b]	100、482、514	3
Our Method	89、196、227、319、324、364、419、517	8

Supplementary Figures

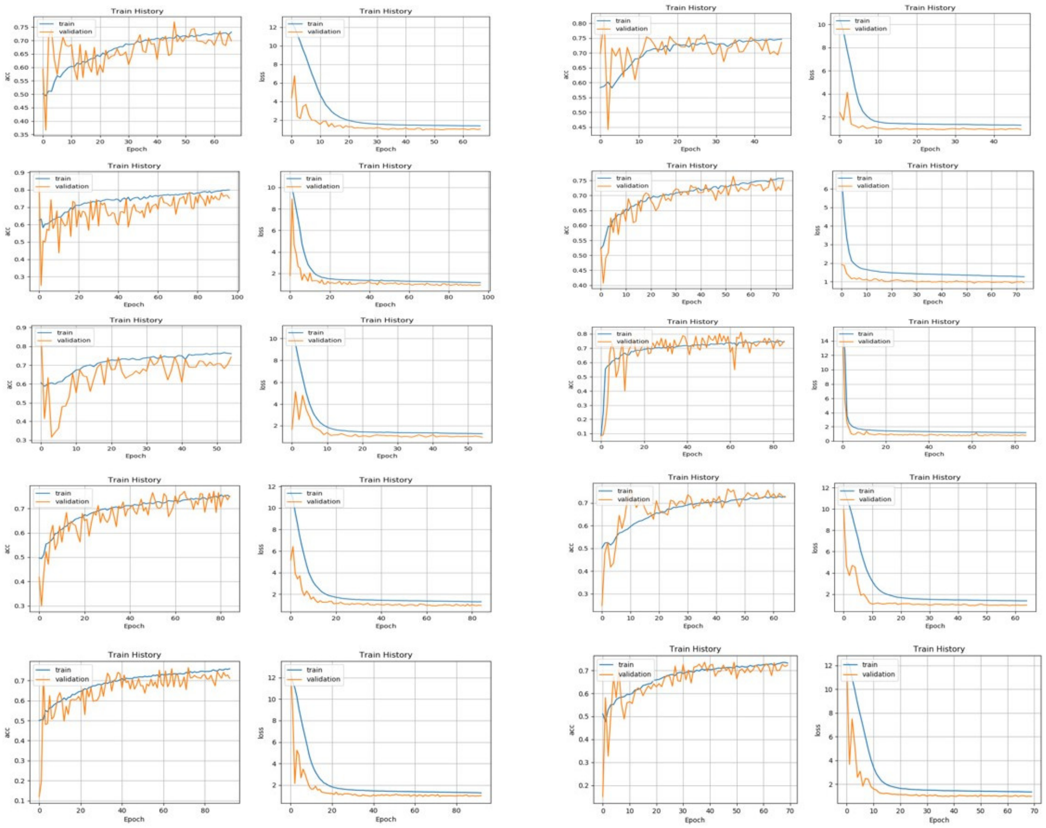

Figure S1. The training/validation loss/accuracy curve of the proposed model MDCAN-Lys by 10-fold cross-validation with window size = 33 on the training set described in Table 1. The blue line is training and the orange line is validation.
